# Surgical outcomes of transverse acetabular fractures and risk factors for poor outcomes

**DOI:** 10.1186/s12891-021-04082-2

**Published:** 2021-03-01

**Authors:** Jae Hoon Jang, Nam Hoon Moon, Seung Joon Rhee, Seok Jin Jung, Tae Young Ahn

**Affiliations:** 1 Department of Orthopedic Surgery, Bio-medical Research Institute, Trauma Center, Pusan National University Hospital, 179 Gudeok-Ro Seo-Gu, Busan, 49241 Republic of Korea; 2 Department of Orthopedic Surgery, Bio-medical Research Institute, Pusan National University Hospital, 179 Gudeok-Ro Seo-Gu, Busan, 49241 Republic of Korea

**Keywords:** Transverse acetabular fracture, Surgical outcome, Prognostic factor, Dome impaction, Residual gap, Residual step

## Abstract

**Background:**

Transverse acetabular fractures, although classified as elementary, have worse outcomes than other types of acetabular fractures. Prognostic factors for this fracture type are not clearly established. This study aimed to assess the surgical outcomes of transverse acetabular fractures and subtypes thereof and to investigate the prognostic factors.

**Methods:**

Between 2014 and 2019, 39 patients (39 hips) had transverse fractures or subtypes thereof. We reviewed the surgical outcomes and evaluated patient factors, injury factors, and surgical factors in relation to osteoarthritis (OA) and conversion to total hip arthroplasty (THA). Additionally, we analyzed the cutoff values for postoperative residual gaps and steps.

**Results:**

Twenty-three male patients and sixteen female with a mean age of 41.7 years (range, 18–78 years) were included. There were 29 satisfactory reductions (74.4%). Eleven hips (28.2%) developed OA, and five (12.8%) of them underwent THA. Dome impaction (odds ratio [OR], 41.173; 95% confidence interval [CI], 1.804–939.814; *p* = 0.020) and residual gaps (OR, 4.251; 95% CI, 1.248–14.479; *p* = 0.021) were correlated with poor outcomes. Residual gaps (≥3 mm) and residual steps (≥1 mm) were significantly associated with OA.

**Conclusions:**

Relatively poor reduction was found for transverse acetabular fractures and subtypes thereof. However, the rates of OA and conversion to THA were not high. Dome impaction and wide residual gaps were identified as risk factors for poor outcomes. The development of OA significantly increased if residual gap and step were more than 3 mm and 1 mm, respectively.

## Background

Although transverse acetabular fractures were classified as an elementary fracture type by Judet and Letournel [1, 2], their anatomical reduction and stable fixation, which are mandatory for surgical treatment of articular fractures, are not easy to achieve [3]. Furthermore, this fracture type is frequently associated with the posterior wall (PW) and caudally oriented (T-component), and these associated patterns are more difficult to manage surgically. Therefore, surgical outcomes of transverse acetabular fractures are considered to be worse than those of other types [4–7].

There have been many reports on the surgical outcomes and prognostic factors of all acetabular fracture types. However, to our knowledge, limited studies have examined the surgical outcomes and prognostic factors of transverse acetabular fractures and subtypes of these fractures. This study aimed to verify the surgical outcomes of the transverse acetabular fracture and its subtypes and to investigate prognostic factors for poor surgical outcomes.

## Methods

### Study population

This retrospective study was approved by our institutional review board. All methods were conducted in accordance with relevant guidelines and regulations. We reviewed the medical records and radiographs of 158 skeletally mature patients who underwent open reduction and internal fixation (ORIF) for an acetabular fracture at a level 1 trauma center between March 2014 and March 2019. Patients with an acetabular fracture including a transverse fracture with or without a PW and/or T-component fracture were eligible for inclusion. The exclusion criteria were having the other type of acetabular fracture, participation in < 12 months of follow-up (except for early conversion to total hip arthroplasty [THA]), incomplete medical records or radiographs, periprosthetic fracture, preexisting hip disease, and open pelvic fracture.

### Surgical procedures and postoperative protocol

All surgeries were performed by a single surgeon trained in the field of orthopedic trauma. Surgical timing was determined based on the patient’s condition and associated injuries. The anterior intrapelvic (modified Stoppa) approach [8] with or without the lateral window of the ilioinguinal approach in the supine position and/or the Kocher-Langenbeck approach in the lateral decubitus position was chosen as the surgical approach based on the configuration of the fracture, the site of the main displacement, and consideration of the associated pelvic ring injury (PRI). The decision to fix the single-column or the double-column, how to perform fixation, and the sequence of reduction and fixation were determined at the surgeon’s discretion based on the fracture configuration, the site of the major fracture, reduction quality, associated PRI, and surgical approach. All implants used for fixation were 3.5 reconstruction plates (titanium; DePuy Synthes, Oberdorf, Switzerland) and/or 3.5 low-profile pelvic plates (stainless steel; DePuy Synthes).

Serial radiographs including pelvic series (anteroposterior, iliac/obturator oblique, and inlet/outlet views) were obtained immediately after surgery and periodically during follow-up. After the drain was removed within 48–72 h after surgery, computed tomography (CT) scans including axial, coronal, and sagittal views were obtained to evaluate the reduction quality and implant positions in all patients. Passive or active range of motion exercises were encouraged as tolerated within the first 2 to 3 days after surgery. Partial weight-bearing with crutches or a walker and full weight-bearing were allowed at 6 to 8 weeks and 12 weeks after surgery, respectively, depending on the patient’s general condition and associated injuries.

### Assessment of measurements

Measurements of demographic data, fracture configurations, and surgical factors and outcomes were recorded. Demographic data included age, sex, body mass index (BMI), American Society of Anesthesiologists classification, diabetes mellitus, smoking history, injury mechanism, injury severity score, and injury side (right or left). Fracture configurations included the plane of the transverse fracture (infratectal, juxtatectal, or transtectal), dome impaction, PW involvement, PW comminution, PW impaction, associated fracture of the T-component, femoral head injury, hip dislocation at the time of the initial injury, and association of the displaced PRI. Surgical factors included the surgical approach, single-column or double-column fixation, postoperative residual gap and step, and classification of reduction quality according to Matta’s criteria [4]. Surgical outcomes included time to union, postoperative infection, nerve injury, heterotopic ossification, osteoarthritis (OA), osteonecrosis of the femoral head (ONFH), and conversion to THA.

The plane of the transverse fracture was classified as follows: infratectal, the main fracture line was oriented across the acetabular fossa; juxtatectal, at the transition of the acetabular fossa to the cranial/superior joint surface; and transtectal, across the superior dome of the acetabulum. PW comminution was defined as a fracture with three or more separate articular fragments. Fracture union was defined as the absence of a fracture line and/or bridging callus across fracture sites on follow-up radiographs of the pelvic series and the ability to perform full weight-bearing ambulation without joint pain and progressive loss of reduction.

Two orthopedic surgeons who did not participate in surgery and were blinded to the surgical outcomes independently measured the postoperative residual gap and step using a standardized CT-based method on a picture archiving and communication system [9, 10]. The averages of each value were used for analyses. Reduction quality was classified as anatomical (≤1 mm), imperfect (1–3 mm), or poor (> 3 mm). The other demographic data, fracture configurations, surgical factors, and surgical outcomes were assessed and documented by one of the authors.

Demographic data, fracture configurations, and surgical factors and outcomes were verified. Additionally, OA and/or ONFH considered poor according to Matta’s grading system [4] and required conversion to THA indicated poor outcomes. Risk factors for the outcomes and cutoff points of related variables were analyzed.

### Statistical analysis

Interobserver reliability of the residual gap and step measurements was evaluated by the intraclass correlation coefficient (ICC). Univariate and multivariate logistic regression tests were performed to determine risk factors for postoperative OA. A receiver-operating characteristic (ROC) curve analysis was used to identify cutoff points for factors that affect OA. MedCalc software (version 18.11; MedCalc Software, Ostend, Belgium) was used for the ROC curve analysis, and SPSS software (version 22.0; SPSS Inc., Chicago, IL, USA) was used for other statistical analyses. Statistical significance was set at *p* < 0.05.

## Results

**Fig. 1:**
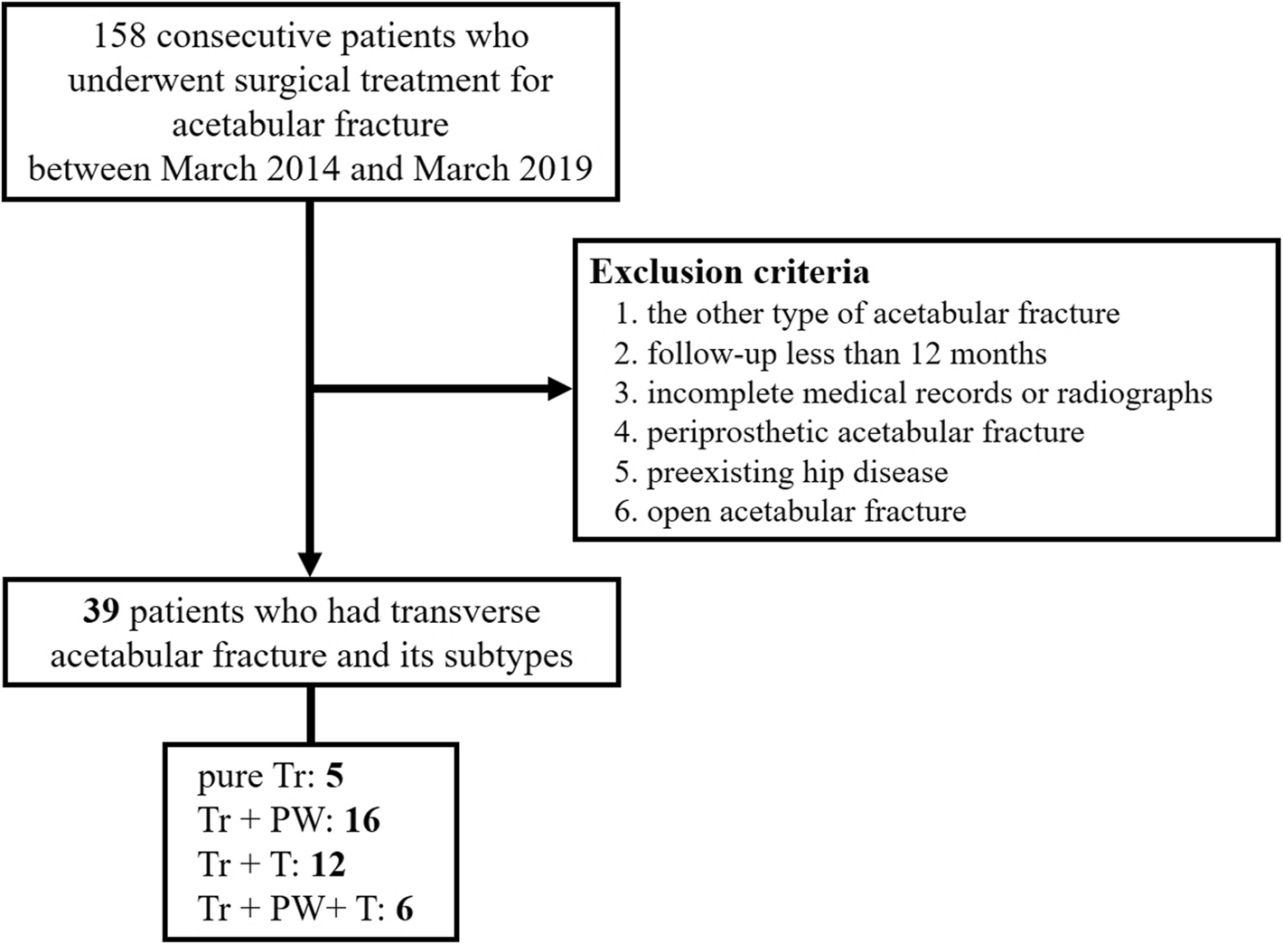
Flowchart of study enrollment. Tr, transverse; PW, posterior wall; T, T-component

Thirty-nine patients (twenty-three men and sixteen women) met the inclusion criteria and had unilateral acetabular fractures (Fig. 1). The mean age and BMI were 41.7 years (range, 18–78 years) and 23.0 kg/m^2^ (range, 16.2–31.3 kg/m^2^), respectively. There were 12 falls from a height, 26 motor vehicle accidents, and one crushing injury. The mean injury severity score was 28.6 (range, 4–66). There were 24 (61.5%) transverse fractures in the juxtatectal region and 15 (38.5%) in the transtectal region. Of all patients, 10 dome impactions (25.6%), 22 PW fractures (56.4%), 12 T-fractures (30.8%), and 17 displaced PRI (43.6%) were associated with the injury. Of the 22 PW fractures, there were 17 cases (43.6%) of comminution and four cases (10.3%) of marginal impaction. Six (15.4%) femoral head injuries and five (12.8%) hip dislocations were observed at the time of initial injury (Table 1).

**Table 1:** Preoperative demographic data and fracture configurations

Number	39
Age (years)	41.7 ± 18.3 (18–78)
Sex (male)	23 (59.0)
Body mass index (kg/m^2^)	23.0 ± 3.4 (16.2–31.3)
ASA physical status classification	
2	16 (41.0)
3	22 (56.4)
4	1 (2.6)
DM	2 (5.1)
Smoking	11 (28.2)
Injury severity score	28.6 ± 14.4 (4–66)
Injury Mechanism	
Fall from height	12 (30.8)
Motor vehicle accident	26 (66.7)
Cushing	1 (2.6)
Side (right)	17 (43.6)
Fracture plane	
Juxtatectal	24 (61.5)
Transtectal	15 (38.5)
Dome impaction	10 (25.6)
Posterior wall involvement	22 (56.4)
Posterior wall comminution	17 (43.6)
Posterior wall impaction	4 (10.3)
Associated T-component	12 (30.8)
Femoral head injury	6 (15.4)
Hip dislocation	5 (12.8)
Association of displaced pelvic ring injury	17 (43.6)

Values are presented as mean ± standard deviation (range), or number (%)

*ASA* American Society of Anesthesiologists, *DM* Diabetes mellitus

ORIF for the fractures was performed after an average of 6.5 days (range, 1–17 days). The Kocher-Langenbeck approach was used for 17 cases, the anterior intrapelvic approach for 18 cases, and both approaches for four cases chosen for the ORIF approach. No patient underwent digastric osteotomy when using the Kocher-Langenbeck approach. Single-column fixation was performed in 18 cases (46.2%). Double-column fixation was performed in 21 cases (53.8%). Among them, a percutaneous technique (retrograde fixation using a 3.5-mm cortical screw for the anterior column) was performed in two cases and ORIF using a screw or plate for the opposite column was performed in the remaining 19 cases. The mean residual gap and step after ORIF were 2.3 mm (range, 0.0–7.0 mm) and 0.6 mm (range, 0.0–4.0 mm), respectively, and there were 14 anatomical (35.9%), 15 imperfect (38.5%), and 10 poor (25.6%) reductions. The ICC was 0.868 (95% confidence interval [CI], 0.747–0.931) for the residual gap and 0.881 (95% CI, 0.773–0.938) for the residual step.

The mean follow-up period was 23.2 months (range, 12.0–51.5 months). An average of 4.7 months (range, 2.1–8.3) was required for fracture union, and there were no cases of non-union. Three patients (7.7%) had postoperative infections that required surgical debridement. Two patients (5.1%) experienced a neurologic deficit of the peroneal part of the sciatic nerve after injury. An iatrogenic injury of the lateral cutaneous femoral nerve occurred in one patient (2.6%) postoperatively. All patients recovered from nerve injuries within 1 year. Sixteen patients (41.0%) had heterotopic ossifications, including eight cases categorized as class I, three as class II, four as class III, and one as class IV according to Brooker classifications [11]. OA was diagnosed in 11 cases (28.2%) during follow-up and in 2 cases (5.1%) accompanied by ONFH. Five (12.8%) of the eleven cases with OA underwent THA (Table 2).

**Table 2:** Surgical factors and outcomes

Time from injury to ORIF (days)	6.5 ± 3.4 (1–17)
Approach	
Kocher-Langenbeck	17 (43.6)
Anterior intrapelvic (±lateral window)	18 (46.2)
Both	4 (10.3)
Fixation	
Single column	18 (46.2)
Double column	21 (53.8)
Residual gap (mm)	2.3 ± 1.5 (0.0–7.0)
Residual step (mm)	0.6 ± 1.2 (0.0–4.0)
Quality of reduction	
Anatomical	14 (35.9)
Imperfect	15 (38.5)
Poor	10 (25.6)
Time to union (months)	4.7 ± 1.6 (2.1–8.3)
Osteoarthritis	11 (28.2)
Osteonecrosis of femoral head	2 (5.1)
Conversion to total hip arthroplasty	5 (12.8)
Follow-up period	23.2 ± 12.3 (12.0–51.5)

Values are presented as mean ± standard deviation (range), or number (%)

*ORIF* Open reduction and internal fixation

In the unadjusted model with the univariate analysis, age (odds ratio [OR], 1.054; 95% CI, 1.009–1.101), BMI (OR, 1.302; 95% CI, 1.018–1.666), dome impaction (OR, 14.583; 95% CI, 2.623–81.084), PW comminution (OR, 4.800; 95% CI, 1.034–22.293), residual gap (OR, 3.612; 95% CI, 1.549–8.420), and residual step (OR, 2.846; 95% CI, 1.346–6.021) were significantly associated with poor outcomes. However, the adjusted model with the multivariate analysis showed that dome impaction (OR, 41.173; 95% CI, 1.804–939.814) and residual gap (OR, 4.251; 95% CI, 1.248–14.479) were significantly associated with poor outcomes (Table 3).

**Table 3:** Binary logistic regression analysis of factures associated with postoperative osteoarthritis in transverse acetabular fractures

	Univariate		Multivariate	
	Unadjusted OR (95% CI)	***P*** value	Adjusted OR (95% CI)	***p*** value
**Patient factors**				
Age	1.054 (1.009–1.101)	0.018	1.066 (0.974–1.166)	0.164
Gender				
Male	Reference			
Female	1.288 (0.315–5.267)	0.725		
Body mass index	1.302 (1.018–1.666)	0.035	1.114 (0.694–1.790)	0.655
Smoking	0.938 (0.197–4.460)	0.935		
**Injury factors**				
Injury severity score	0.950 (0.896–1.008)	0.089		
Fracture plane				
Juxtatectal	Reference			
Transtectal	2.533 (0.608–10.559)	0.202		
Dome impaction	14.583 (2.623–81.084)	0.002	41.173 (1.804–939.814)	0.020
Posterior wall involvement	3.150 (0.738–13.448)	0.121		
Posterior wall comminution	4.800 (1.034–22.293)	0.045	17.401 (0.685–442.182)	0.084
Posterior wall impaction	10.125 (0.922–111.247)	0.058		
Associated T-component	0.792 (0.169–3.714)	0.767		
Femoral head injury	3.125 (0.523–18.669)	0.212		
Hip dislocation	1.852 (0.265–12.947)	0.535		
Association of displaced pelvic ring injury	1.111 (0.273–4.520)	0.883		
**Surgical factors**				
Time from injury to ORIF	1.162 (0.941–1.434)	0.163		
Approach				
Kocher-Langenbeck	Reference			
Anterior intrapelvic (±lateral window)	0.524 (0.118–2.327)	0.395		
Both	0.611 (0.052–7.240)	0.611		
Fixation				
One-column	Reference			
Two-column	1.040 (0.256–4.218)	0.956		
Residual gap	3.612 (1.549–8.420)	0.003	4.251 (1.248–14.479)	0.021
Residual step	2.846 (1.346–6.021)	0.006	1.749 (0.556–5.499)	0.339

*p* value < 0.05 by univariate analysis were included in the multivariate logistic regression analysis

*CI* Confidence interval, *OR* Odds ration, *ORIF* Open reduction and internal fixation

**Fig. 2:**
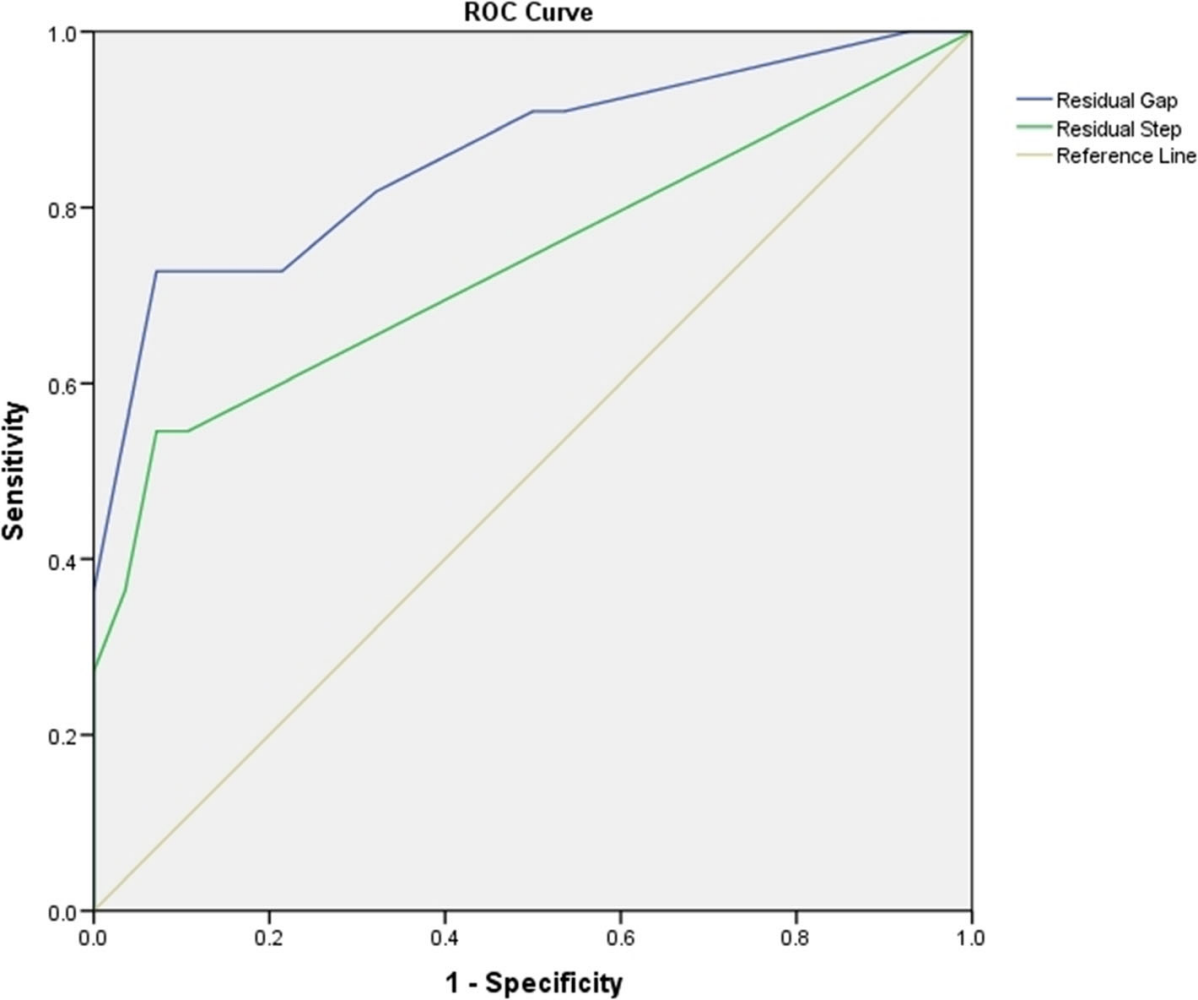
Receiver-operating characteristic curves for residual gap and step displacement associated with poor outcomes. The diagonal line is the reference line. The residual gap is shown in blue, and residual step displacement is shown in green

ROC curves showed areas under the curve of 0.859 (95% CI, 0.710–0.949; *p* < 0.0001) for the residual gap and 0.737 (95% CI, 0.572–0.865; *p* = 0.0062) for the residual step (Fig. 2). A residual gap of 3.0 mm (sensitivity, 72.73%; specificity, 92.86%) and residual step of 1.0 mm (sensitivity, 54.55%; specificity, 92.86%) were proven to be significant cutoff points for poor outcomes (Table 4).

**Table 4:** Receiver-operating characteristic curves analysis for cutoff points of residual gap and step

	AUC (95% CI)	Cutoff points	Sensitivity (95% CI)	Specificity (95% CI)	***p*** value
Residual gap (mm)	0.859 (0.710–0.949)	3.0	72.73 (39.0–94.0)	92.86 (76.5–99.1)	< 0.0001
Residual step (mm)	0.737 (0.572–0.865)	1.0	54.55 (23.4–83.3)	92.86 (76.5–99.1)	0.0062

The cutoff points were selected using Youden index J

*AUC* Area under the receiver-operating characteristic curve, *CI* Confidence interval

## Discussion

We included pure transverse, transverse and PW, and T-type acetabular fractures in this study; furthermore, transverse fractures and their subtypes have been reported to account for as little as 10% and as much as 40% of all acetabular fractures [2, 4, 10, 12–15]. However, few studies have focused on only these fractures as it is challenging to obtain anatomical reduction and stable fixation and often result in worse surgical outcomes than other types [3–5]. Therefore, we sought to examine the surgical outcomes of and prognostic factors for poor outcomes of these acetabular fracture types. In this study, 39 (24.7%) of the 158 patients who underwent ORIF for an acetabular fracture had a transverse acetabular fracture or a subtype (pure transverse fractures, 5 cases [12.8%]; PW fractures, 16 cases [41.0%]; T-component fractures, 12 cases [30.8%]; PW and T-component fractures, 6 cases [14.4%]). Fourteen anatomical (35.9%), 15 imperfect (38.5%), and 10 poor (25.6%) reductions were obtained postoperatively. Eleven cases (28.2%) had poor grades according to Matta’s grading system [4], and age, BMI, dome impaction, PW comminution, and residual gap and step were risk factors for these poor outcomes according to the univariate analysis. However, dome impaction and residual gap were identified as significant risk factors in the multivariate analysis. Furthermore, a residual gap more than 3.0 mm and residual step more than 1.0 mm were significant predictors of poor outcomes according to the ROC curve analysis.

**Fig. 3:**
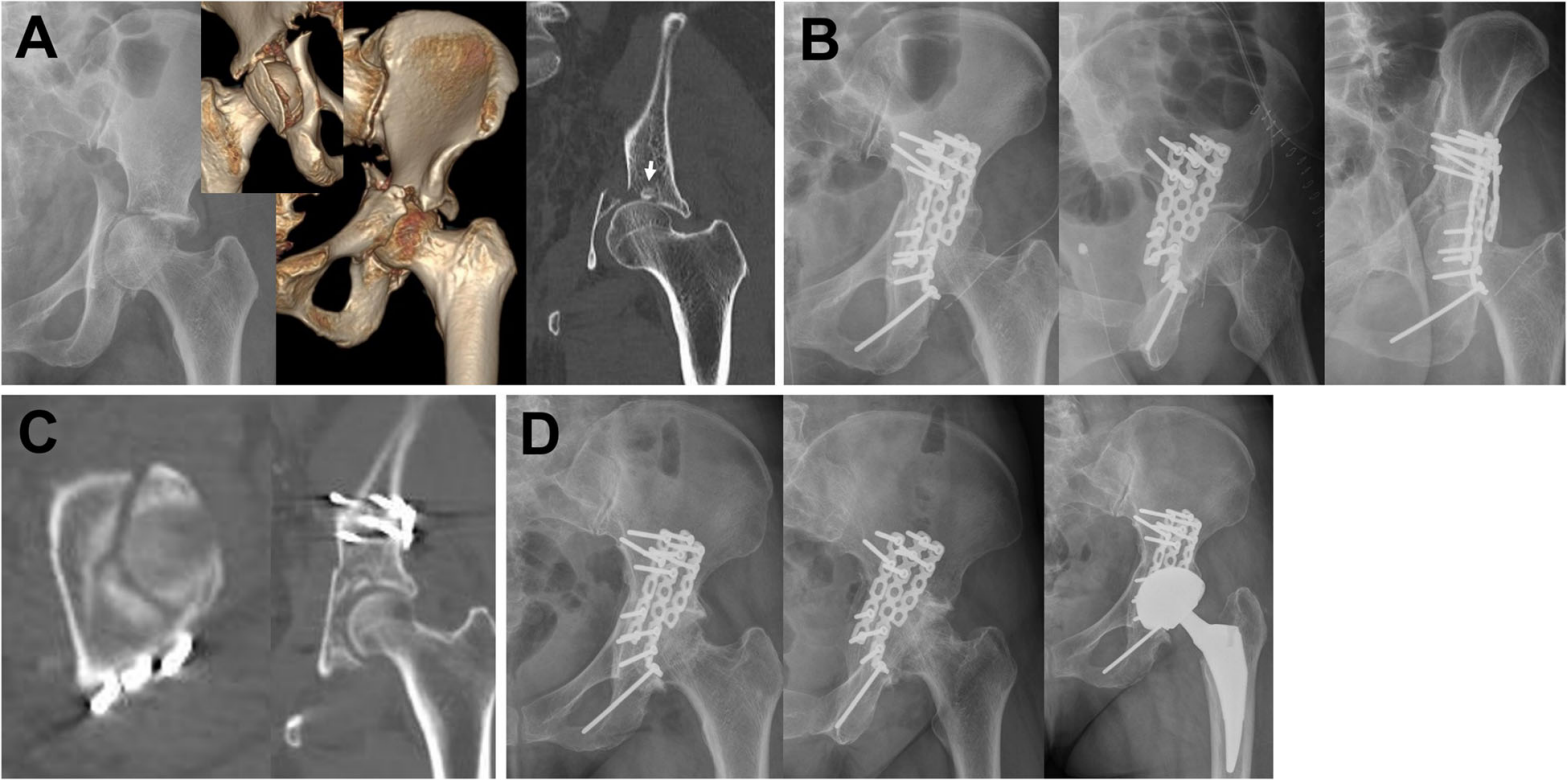
A 47-year old woman involved in a traffic accident. **a** Immediate radiograph and three-dimensional computed tomography (CT) images show transverse and posterior wall fractures of acetabulum. Dome impaction (arrow) was observed in coronal view of CT scan. **b** Postoperative radiographs revealed that satisfactory reduction and fixation were achieved. **c** However, axial and coronal CT views show a residual gap > 3 mm and poor reduction and fixation of the dome fragment. **d** Osteoarthritis developed and total hip arthroplasty was performed at 6 months after surgery

Previous studies have reported satisfactory reduction rates from 57 to 100% [5–7, 12, 14, 16, 17]. The current study showed a satisfactory reduction rate of 74.4%, which was worse than that of studies that examined the rate based on plain radiographs. However, Frietman et al. found worse results (57%) when measuring reduction quality using CT [12]. We believe that the accuracy of the assessment modality could explain these differences. Reduction quality was more apparent and could be classified more precisely using CT images compared to plain radiographs, which yielded a low rate of satisfactory reduction in our study [5, 9]. Posttraumatic OA was found in 20–40% of patients with an acetabular fracture and in 9–35% of patients undergoing THA [5, 7, 10, 12, 14, 18]. The current study found that 11 cases (28.2%) developed posttraumatic OA and five cases (12.8%) underwent THA, which is similar to previous studies [5–7, 12, 14, 17]. In most studies, unsatisfactory Merle d’Aubigné and Postel scores or THA conversion indicated poor outcomes [5, 7, 10, 12, 18]. Based on these criteria, risk factors for poor outcomes included old age (age 50 years or older), overweight, high-energy trauma, articular surface comminution or impaction, large initial displacement, femoral head dislocation, residual gap, residual step, and even surgeon training [10, 12, 13, 19–22]. However, the functional score has a limited ability to represent patients’ daily functions [12, 23], and THA is the end result of OA. Therefore, we considered OA and/or conversion to THA as indicators of poor outcomes, and residual gap and dome impaction were identified as significant risk factors in the multivariate analysis (Table 5, Fig. 3).

**Table 5:** Comparison of results with previous studies

Author	Age	Enrolled type of acetabular fracture	Number	Satisfactory reduction	OA	THA conversion	Poor outcome setting	Risk factors for poor outcome
Mears et al. [5]	46.5	All types	424	372 (88%)	98 (23%)	48 (11%)	Low Harris hip score	N/A
Dunet et al. [16]	41.6	All types	72	N/A	29 (40%)	25 (35%)	Unsatisfactory MDP	Overweight, road accident, posterior wall fracture, initial intraarticular foreign body, unsatisfactory reduction
Meena et al. [7]	38.8	All types	118	110 (93%)	34 (29%)	10 (9%)	Unsatisfactory MDP	Femoral head dislocation, initial displacement (> 20 mm), associated injury, delay in surgery (> 14 days)
Frietman et al. [10]	48.5	All types	220	125 (57%)	55 (25%)	33 (15%)	THA conversion	Old age, marginal impaction, extended iliofemoral approach, unsatisfactory reduction
Verbeek et al. [9]	51.2	All types	227	N/A	N/A	55 (24%)	THA conversion	Age ≥ 50 yr, gap ≥5 mm, step ≥1 mm in young patients
Oh et al. [6]	46.6	Tr ± PW	15	11 (69%)	2 (27%)	N/A	Unsatisfactory MDP	Gap > 2 mm, dome comminution, femoral head cartilage injury
Li et al. [15]	34.0	Transtectal Tr	37	35 (95%)	N/A	N/A	Unsatisfactory MDP	Acetabular roof comminution
Masse et al. [12]	35.3	Tr ± PW or T	31	25 (81%)	4 (13%)	4 (13%)	N/A	N/A
Fahmy et al. [14]	31.0	Tr ± PW	30	30 (100%)	3 (10%)	N/A	N/A	N/A
Current study	41.7	Tr ± PW and/or T	39	29 (74.4%)	11 (28.2%)	5 (12.8%)	OA ± ONFH THA conversion	Residual gap, dome impaction

*AFI* Acetabular facture index, *MDP* Merle d’Aubigné and Postel score, *N/A* Not available, *OA* Osteoarthritis, *ONFH* Osteonecrosis of femoral head, *PW* Posterior wall, *T* T-component, *THA* Total hip arthroplasty, *Tr* Transverse

**Fig. 4:**
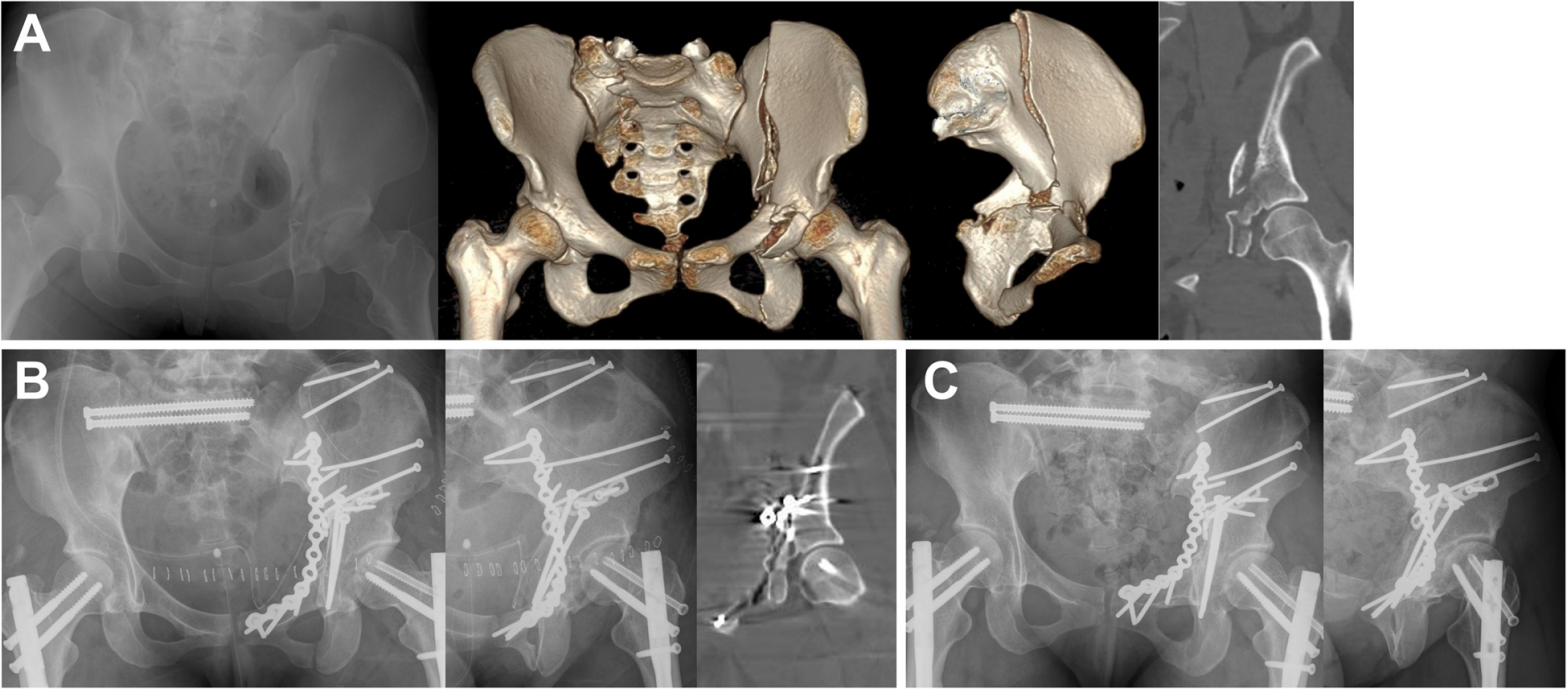
A 23-year old woman attempted suicide by jumping from a height. **a** The patient sustained a T-type fracture of the acetabulum without dome involvement and displaced pelvic ring injury. **b** Postoperative radiographs and coronal view of CT scan after open reduction and internal fixation for the acetabular fracture and pelvic ring injury are shown. The fractures were reduced and fixed via the anterior intrapelvic approach due to the associated iliac bone fracture and no involvement of the posterior wall. **c** Radiographs at 12 months after surgery show bony union and no evidence of arthritic change

Previous studies reported that 27–30% of acetabular fractures were associated with PRI [7, 20]. However, 17 (43.6%) of 39 cases were associated with PRI in this study. Although it is not clear whether more PRI are accompanied by transverse acetabular fractures compared with other acetabular fracture types, this study included only transverse acetabular fractures and subtypes, which are commonly caused by lateral compression; furthermore, lateral compression is one of the main mechanisms of PRI. Additionally, this study was conducted at a level 1 territory trauma center. Most patients were injured by high-energy trauma, which results in complex injuries. When the PRI is associated with an acetabular fracture, anatomical restoration of the articular surface is difficult due to disruption of the pelvic ring structure and the prognosis is poorer [2]. To overcome this difficulty, reduction was performed from the site where it is easier to obtain more accurate reduction to the site where it is more difficult according to the PRI configuration. Fixation was completed by repeating partial fixation sequentially and alternately without immediate firm fixation for each site. Through this process, we could create a proper environment to obtain accurate reduction of the acetabular fracture. We believe these attempts might reduce the negative effects of PRI. Statistical significance was not identified as a risk factor (Fig. 4).

A single surgeon at a single institution performed all surgeries, which is a strength of this study that could have reduced bias. However, this study had several limitations, including a retrospective design and small sample size, which could have generated an underpowered analysis. Patients with high-energy trauma had various conditions and associated injuries that acted as confounding variables. Therefore, clinical results and functional scores that are significant to the prognosis of acetabular fractures were omitted from this study. To obtain more accurate results, a large multicenter study that includes additional variables should be performed.

## Conclusion

This study showed a relatively low rate of satisfactory reduction based on CT measurements of the transverse acetabular fracture and its subtypes. However, the rates of OA and conversion to THA were not high. Dome impaction and a wide residual gap were identified as risk factors for poor outcomes, and the development of OA significantly increased if the residual gap and step were more than 3 mm and 1 mm, respectively. Poorer surgical outcomes and specific prognostic factors were not found for these fractures. Anatomical reduction is mandatory to optimize surgical outcomes, especially when dome impaction is involved.

## Data Availability

The datasets analyzed during the current study are not publicly available due to the health policy of protection of patient privacy announced by the Ministry of Health and Welfare of Korea but are available from the corresponding author on reasonable request.

## Abbreviations

BMI: Body mass index
CI: Confidence interval
CT: Computed tomography
OA: Osteoarthritis
ONFH: Osteonecrosis of the femoral head
OR: Odds ratio
ORIF: Open reduction and internal fixation
PRI: Pelvic ring injury
PW: Posterior wall
ROC: Receiver-operating characteristic
THA: Total hip arthroplasty

## References

[1] Letournel E (1980). Acetabulum fractures: classification and management. Clin Orthop Relat Res.

[2] Letournel E, Judet R (1993). Fractures of the acetabulum.

[3] Byun S, Mauffrey C, Yoo J, Park C, Hwang J (2019). Simulation for reduction of transverse acetabular fractures in sawbones models. J Korean Fract Soc.

[4] Matta JM (1996). Fractures of the acetabulum: accuracy of reduction and clinical results in patients managed operatively within three weeks after the injury. J Bone Joint Surg Am.

[5] Mears DC, Velyvis JH, Chang CP (2003). Displaced acetabular fractures managed operatively: indicators of outcome. Clin Orthop Relat Res.

[6] Oh CW, Kim PT, Park BC (2006). Results after operative treatment of transverse acetabular fractures. J Orthop Sci.

[7] Meena UK, Tripathy SK, Sen RK, Aggarwal S, Behera P (2013). Predictors of postoperative outcome for acetabular fractures. Orthop Traumatol Surg Res..

[8] Cole JD, Bolhofner BR (1994). Acetabular fracture fixation via a modified Stoppa limited intrapelvic approach. Description of operative technique and preliminary treatment results. Clin Orthop Relat Res.

[9] Verbeek DO, van der List JP, Villa JC, Wellman DS, Helfet DL (2017). Postoperative CT is superior for acetabular fracture reduction assessment and reliably predicts hip survivorship. J Bone Joint Surg Am.

[10] Verbeek DO, van der List JP, Tissue CM, Helfet DL (2018). Predictors for long-term hip survivorship following acetabular fracture surgery: importance of gap compared with step displacement. J Bone Joint Surg Am.

[11] Brooker AF, Bowerman JW, Robinson RA, Riley LH (1973). Ectopic ossification following total hip replacement. Incidence and a method of classification. J Bone Joint Surg Am.

[12] Frietman B, Biert J, Edwards MJR (2018). Patient-reported outcome measures after surgery for an acetabular fracture. Bone Joint J.

[13] Giannoudis PV, Grotz MR, Papakostidis C, Dinopoulos H (2005). Operative treatment of displaced fractures of the acetabulum. A meta-analysis. J Bone Joint Surg Br.

[14] Masse A, Aprato A, Rollero L, Bersano A, Ganz R (2013). Surgical dislocation technique for the treatment of acetabular fractures. Clin Orthop Relat Res.

[15] Mayo KA (1994). Open reduction and internal fixation of fractures of the acetabulum. Results in 163 fractures. Clin Orthop Relat Res.

[16] Fahmy M, Abdel Karim M, Khaled SA, Abdelazeem AH, Elnahal WA, Elnahal A (2018). Single versus double column fixation in transverse fractures of the acetabulum: a randomised controlled trial. Injury..

[17] Li XG, Tang TS, Sun JY (2010). Results after surgical treatment of transtectal transverse acetabular fractures. Orthop Surg.

[18] Dunet B, Tournier C, Billaud A, Lavoinne N, Fabre T, Durandeau A (2013). Acetabular fracture: long-term follow-up and factors associated with secondary implantation of total hip arthroplasty. Orthop Traumatol Surg Res.

[19] Boudissa M, Ruatti S, Kerschbaumer G, Milaire M, Merloz P, Tonetti J (2016). Part 2: outcome of acetabular fractures and associated prognostic factors-a ten-year retrospective study of one hundred and fifty six operated cases with open reduction and internal fixation. Int Orthop.

[20] Gänsslen A, Hildebrand F, Kretek C (2013). Transverse + posterior wall fractures of the acetabulum: epidemiology, operative management and long-term results. Acta Chir Orthop Traumatol Cechoslov.

[21] Long HT, Deng ZH, Zou M, Lin ZY, Zhu JX, Zhu Y (2017). Effects of the acetabular fracture index and other factors of posterior wall acetabular fracture on functional outcome. J Int Med Res.

[22] Moed BR, McMahon MJ, Armbrecht ES (2016). The acetabular fracture prognostic nomogram: does it work for fractures of the posterior wall?. J Orthop Trauma.

[23] Moed BR, McMichael JC (2007). Outcomes of posterior wall fractures of the acetabulum. J Bone Joint Surg Am.

